# Carrier Status for the Common R501X and 2282del4 Filaggrin Mutations Is Not Associated with Hearing Phenotypes in 5377 Children from the ALSPAC Cohort

**DOI:** 10.1371/journal.pone.0005784

**Published:** 2009-06-03

**Authors:** Santiago Rodriguez, Amanda J. Hall, Raquel Granell, W. H. Irwin McLean, Alan D. Irvine, Colin N. A. Palmer, George Davey Smith, John Henderson, Ian N. M. Day

**Affiliations:** 1 Bristol Genetic Epidemiology Laboratories (BGEL), Department of Social Medicine, University of Bristol, Bristol, United Kingdom; 2 MRC Centre for Causal Analyses in Translational Epidemiology (CAiTE), Department of Social Medicine, University of Bristol, Bristol, United Kingdom; 3 Centre for Hearing and Balance Studies, University of Bristol, Bristol, United Kingdom; 4 Department of Social Medicine, University of Bristol, Bristol, United Kingdom; 5 Epithelial Genetics Group, Human Genetics Unit, Division of Pathology and Neuroscience, University of Dundee, Ninewells Hospital and Medical School, Dundee, United Kingdom; 6 Department of Paediatric Dermatology, Our Lady's Hospital for Sick Children, Dublin, Ireland; 7 Population Pharmacogenetics Group, Biomedical Research Centre, University of Dundee, Ninewells Hospital and Medical School, Dundee, United Kingdom; 8 Department of Community Based Medicine, University of Bristol, Bristol, United Kingdom; Stanford University, United States of America

## Abstract

**Background:**

Filaggrin is a major protein in the epidermis. Several mutations in the filaggrin gene (*FLG*) have been associated with a number of conditions. Filaggrin is expressed in the tympanic membrane and could alter its mechanical properties, but the relationship between genetic variation in *FLG* and hearing has not yet been tested.

**Methodology/Principal Findings:**

We examined whether loss-of function mutations R501X and 2282del4 in the *FLG* gene affected hearing in children. Twenty eight hearing variables representing five different aspects of hearing at age nine years in 5,377 children from the Avon Longitudinal Study of Parents and Children (ALSPAC) cohort were tested for association with these mutations. No evidence of association was found between R501X or 2282del4 (or overall *FLG* mutation carrier status) and any of the hearing phenotypes analysed.

**Conclusions/Significance:**

In conclusion, carrier status for common filaggrin mutations does not affect hearing in children.

## Introduction

Filaggrin (filament aggregation protein) is a major protein in the cornified envelope of the epidermis and is involved in maintaining the skin barrier [1], [2]. The filaggrin gene (*FLG*) in humans encodes a polyprotein precursor (profilaggrin) which, after post-translational processing, results in several individual filaggrin polypeptides [3]. Filaggrin has been linked to several clinical phenotypes, with filaggrin mutations being directly associated with the cause, susceptibility to, or modification of the clinical expression of several diseases including dermatological disorders [2]. Common mutations in *FLG* have been implicated in the causation of ichthyosis vulgaris and appear to be a major risk factor for atopic dermatitis, asthma associated with atopic dermatitis, eczema, sensitization to grass, house dust mite and cat dander, and sensitization to multiple allergens [2], [4], [5]. Two of the most studied mutations (R501X and 2282del4) are common in populations of European ancestry [6]. Both R501X and 2282del4 are loss-of function mutations leading to truncation of filaggrin translation by creating premature termination codons [7]. Both mutations have a complex role both in Mendelian disease and in complex diseases. An example is ichthyosis vulgaris. R501X and 2282del4 have been suggested to be the cause of ichthyosis vulgaris in 15 families and isolated cases, acting in a semidominant fashion with incomplete penetrance [7]. Homozygotes for either mutation or compound heterozygous are severely affected, with heterozygotes only mildy affected [7]. Additional reports confirmed the role of R501X and 2282del4 on ichthyosis vulgaris ([8] for review). One of these studies [9] showed that heterozygous individuals were also severely affected, suggesting that additional mutations might be present in these patients.

It has been shown that loss or reduction of filaggrin leads to impaired keratinization and defective skin barrier [7]. In fact, although it has not been pursued in the context of filaggrin mutations, filaggrin is also expressed in the tympanic membrane, where it is involved in the soft keratinization of the epidermal layer [10]. Therefore, FLG mutations could affect hearing. In addition, filaggrin has been localised immunohistochemically in middle ear cholesteatoma in humans [11], this condition leading to an alteration of epidermal differentiation in middle ear [12]. Expression of *FLG* in these locations affecting the cornified envelope of the tympanic epidermis has the potential to influence hearing. The high prevalence of *FLG* mutations could reflect chance, but selective advantage through a trait such as hearing is another possibility. We hypothesized that such changes would affect air but not bone conduction as they affect tympanic membrane and not neural mechanisms.

Clinical evidence available relates impaired keratinization to the filaggrin mutations R501X and 2282del4. To date, there has been no consideration of the possible effects with respect to filaggrin expression in the tympanic membrane. Based on this evidence we tested whether carrier status for a single copy of these mutations is a risk or protective factor for hearing loss/enhanced hearing in an epidemiological survey of 5,377 UK children with detailed phenotypic information for a range of hearing phenotypes.

## Materials and Methods

### Participants

ALSPAC is a longitudinal, population-based birth cohort study that recruited 14,541 pregnant women residing in Avon, United Kingdom, with expected dates of delivery between April 1, 1991, and December 31, 1992. There were 14,062 liveborn children. The study protocol has been described previously [13], [14] and further details are available on the ALSPAC Web site (http://www.bris.ac.uk/alspac). Ethical approval for all aspects of data collection was obtained from the ALSPAC Law and Ethics Committee (institutional review board 00003312). Written informed consent for the study was obtained.

### Genotyping

Genotyping was done as previously described [4]. In brief, R501X and 2282del4 were genotyped with the TaqMan allelic discrimination assays (Applied Biosystems, Foster City, California) in 384-well plates. Double-checking with other technical approaches was performed in a substantial fraction of the samples, including all the identified homozygotes of both variants and all 2282del4 carriers.

### Hearing variables

All study participants were invited to a research clinic at 9 years of age where five different hearing phenotypes were measured: hearing thresholds, transient evoked (TE) otoacoustic emission (OAE) amplitude, middle ear compliance and pressure.

For these phenotypes, a total of 28 variables were analysed: hearing thresholds at five frequencies (0.5, 1, 2, 4 and 8 kHz) for air conduction and three frequencies (0.5, 1, 2 kHz) for bone conduction were measured.

Tympanometry was used to measure middle ear compliance and middle ear pressure. TEOAE were evoked using a click stimulus of approximately 70 dB SPL (linear mode), and the response amplitude obtained for the total response and at frequency bands 1–4 kHz. All measures (except bone conduction thresholds) were obtained for both left and right ears.

Since both mutations are inactivating mutations likely to have similar or identical effects and homozygotes and compound heterozygotes are rare, we also undertook a combined analysis (one degree of freedom) for each variable for *FLG* mutation carrier vs *FLG* mutation non-carrier.

In addition to the analysis of each variable in all individuals, we tested whether each of the two mutations was associated with better versus worse hearing, using a cut off of < = 0 dB, both for hearing thresholds (right and left), and for bone conduction acute hearing thresholds. The effect of both mutations was also analysed by testing the association between each of the mutations and better versus worse cochlear function/middle ear transmission (worse cochlear function defined as the bottom quartile of the TEOAE amplitude distribution).

We tested whether R501X or 2282del4 associated with bilateral middle ear effusion. Finally, we tested whether the filaggrin mutations associated with better hearing thresholds/TEOAE in cases with type A tympanograms (i.e. no middle ear effusion).

### Statistical analyses

The associations between each of the two mutations and continuous variables were tested by means of one-way ANOVA analyses. Analyses involving the discrete variable bilateral middle ear effusion were performed by means of a Pearson χ^2^ contingency test. All analyses were performed in SPSS ver. 15.0.

## Results

Complete data on genotypic data and at least one hearing variable were available for 5,377 children.

There were no significant deviations from Hardy-Weinberg equilibrium for the mutations analysed (data not shown). The allele frequencies observed for R501X (0.021) and 2282del4 (0.023) are consistent with the allele frequencies observed in other studies (ranging from 0,021 to 0.041 for R501X, and from 0.005 to 0.019 for 2282del4) [9].

Tables 1 and 2 show the results from the association tests between each of the 28 variables analysed and R501X and 2282del4 respectively. There was no single nominal association between any of the 28 variables and R501X. For 2282del4, we found a nominal association (P = 0.0057) with air conduction hearing threshold level at 2 kHz on the right ear, but no association with the same hearing threshold for the left ear (P = 0.6855). The audiograms for two children homozygous for 2282del4 (who averaged right ear 2 kHz data which was significant; P = 0.0057) indicated an isolated right ear high threshold (35 dB) for one subject, whereas the other subject and both left ear values were near the population average. No association was found between the 28 variables and overall *FLG* mutation carrier vs non-carrier status (Table 3). The results observed when considering the combined homozygote status association (compound heterozygous for both mutations plus homozygotes for 2282del4) were not remarkable (data not shown).

**Table 1:** Effect of *FLG* R501X on 30 hearing variables as tested by one-way ANOVA tests.

		R501X	N	Mean	SD	F	P
Right ear							
Air conduction hearing threshold (dB HL)	0.5 kHz	AA	4952	6.20	7.24	0.12	0.7328
		Aa	219	6.37	6.75		
	1 kHz	AA	5026	4.38	7.66	0.23	0.6291
		Aa	223	4.13	6.61		
	2 kHz	AA	5025	4.04	7.33	0.47	0.4914
		Aa	223	3.70	5.80		
	4 kHz	AA	5024	3.85	8.34	1.12	0.2908
		Aa	223	3.25	7.90		
	8 kHz	AA	4932	9.05	10.09	0.34	0.5583
		Aa	218	8.65	9.10		
	Average 0.5, 1, 2, 4, 8 kHz	AA	4926	5.47	6.34	0.33	0.5656
		Aa	217	5.22	5.63		
Left ear							
Air conduction hearing threshold (dB HL)	0.5 kHz	AA	4954	6.48	7.30	0.02	0.8952
		Aa	219	6.42	6.57		
	1 kHz	AA	5025	4.52	7.67	2.49	0.1150
		Aa	223	3.70	5.94		
	2 kHz	AA	5023	4.24	7.53	0.76	0.3831
		Aa	223	3.79	6.07		
	4 kHz	AA	5022	4.68	8.60	0.63	0.4289
		Aa	222	4.21	7.10		
	8 kHz	AA	4931	9.16	10.36	0.69	0.4078
		Aa	217	8.57	9.64		
	Average 0.5, 1, 2, 4, 8 kHz	AA	4929	5.78	6.30	1.18	0.2781
		Aa	216	5.31	5.19		
Bone conduction hearing threshold (dB HL)	0.5 kHz	AA	4859	−1.24	6.53	0.06	0.8069
		Aa	215	−1.35	6.02		
	1 kHz	AA	5000	−2.30	6.52	0.07	0.7956
		Aa	222	−2.18	5.81		
	2 kHz	AA	4868	0.95	7.26	0.20	0.6557
		Aa	215	0.72	6.84		
	Average 0.5, 1, 2 kHz	AA	4854	−0.89	5.38	0.01	0.9085
		Aa	215	−0.93	5.01		
Left ear							
TEOAE amplitude (dB SPL)	1 kHz	AA	3865	−8.75	6.96	0.03	0.8677
		Aa	164	−8.66	7.56		
	2 kHz	AA	3899	−10.16	6.74	0.06	0.8092
		Aa	165	−10.29	6.55		
	3 kHz	AA	3893	−12.12	7.14	1.24	0.2661
		Aa	166	−12.75	7.05		
	4 kHz	AA	3906	−13.81	7.57	0.53	0.4680
		Aa	166	−14.24	6.86		
	Total response	AA	3917	9.62	5.69	0.05	0.8229
		Aa	166	9.52	5.36		
Right ear							
TEOAE amplitude (dB SPL)	1 kHz	AA	3704	−7.66	6.91	2.63	0.1048
		Aa	154	−6.73	7.20		
	2 kHz	AA	3765	−9.04	6.52	3.50	0.0615
		Aa	154	−8.04	6.11		
	3 kHz	AA	3761	−11.44	7.18	0.08	0.7736
		Aa	154	−11.27	6.76		
	4 kHz	AA	3757	−13.54	7.42	0.60	0.4401
		Aa	152	−14.01	7.07		
	Total response	AA	3776	10.46	5.65	2.89	0.0894
		Aa	154	11.25	5.46		
Left ear							
Tympanometry	Middle ear compliance (cm^3^)	AA	4785	0.64	0.42	1.72	0.1895
		Aa	208	0.68	0.47		
	Middle ear pressure (daPa)	AA	4796	−39.66	61.80	0.01	0.9430
		Aa	207	−39.35	68.24		
Right ear							
Tympanometry	Middle ear compliance (cm^3^)	AA	4791	0.62	0.42	0.13	0.7215
		Aa	206	0.63	0.43		
	Middle ear pressure (daPa)	AA	4804	−39.47	61.42	0.14	0.7119
		Aa	204	−37.84	64.31		

**Table 2:** Effect of *FLG* 2282del4 on 30 hearing variables as tested by one-way ANOVA tests.

		2282del4	N	Mean	SD	F	P
Right ear							
Air conduction hearing threshold (dB HL)	0.5 kHz	AA	4932	6.19	7.19	0.32	0.7243
		Aa	238	6.55	7.83		
		aa	2	7.50	3.54		
	1 kHz	AA	5010	4.32	7.53	2.29	0.1018
		Aa	238	5.36	9.15		
		aa	2	7.50	3.54		
	2 kHz	AA	5009	4.00	7.20	5.16	0.0057
		Aa	238	4.39	8.43		
		aa	2	20.00	21.21		
	4 kHz	AA	5008	3.80	8.30	0.51	0.5999
		Aa	238	4.35	8.71		
		aa	2	2.50	3.54		
	8 kHz	AA	4915	9.01	10.05	0.32	0.7282
		Aa	234	9.53	10.09		
		aa	2	7.50	3.54		
	Average 0.5, 1, 2, 4, 8 kHz	AA	4908	5.43	6.26	1.40	0.2456
		Aa	234	6.06	7.29		
		aa	2	9.00	4.24		
Left ear							
Air conduction hearing threshold (dB HL)	0.5 kHz	AA	4934	6.48	7.27	0.05	0.9520
		Aa	238	6.37	7.20		
		aa	2	7.50	3.54		
	1 kHz	AA	5009	4.48	7.59	0.03	0.9664
		Aa	238	4.60	8.01		
		aa	2	5.00	7.07		
	2 kHz	AA	5007	4.20	7.44	0.38	0.6855
		Aa	238	4.62	8.19		
		aa	2	5.00	0.00		
	4 kHz	AA	5005	4.67	8.54	0.34	0.7106
		Aa	238	4.50	8.59		
		aa	2	0.00	7.07		
	8 kHz	AA	4913	9.15	10.32	0.10	0.9009
		Aa	234	8.85	10.60		
		aa	2	10.00	0.00		
	Average 0.5, 1, 2, 4, 8 kHz	AA	4910	5.75	6.23	0.01	0.9889
		Aa	234	5.81	6.99		
		aa	2	5.50	3.54		
Bone conduction hearing threshold (dB HL)	0.5 kHz	AA	4840	−1.25	6.45	0.44	0.6461
		Aa	233	−1.05	7.72		
		aa	2	2.50	10.61		
	1 kHz	AA	4984	−2.30	6.39	0.58	0.5610
		Aa	237	−2.19	8.27		
		aa	2	2.50	3.54		
	2 kHz	AA	4849	0.93	7.17	0.34	0.7145
		Aa	233	1.03	8.62		
		aa	2	5.00	7.07		
	Average 0.5, 1, 2 kHz	AA	4835	−0.90	5.27	0.72	0.4870
		Aa	233	−0.74	7.19		
		aa	2	3.33	2.36		
Left ear							
TEOAE amplitude (dB SPL)	1 kHz	AA	3838	−8.75	7.01	0.04	0.9621
		Aa	191	−8.68	6.62		
		aa	1	−7.10	.		
	2 kHz	AA	3871	−10.15	6.73	0.17	0.8443
		Aa	193	−10.44	6.80		
		aa	1	−10.60	.		
	3 kHz	AA	3867	−12.14	7.16	0.06	0.9387
		Aa	192	−12.32	6.63		
		aa	1	−12.70	.		
	4 kHz	AA	3880	−13.82	7.56	0.15	0.8629
		Aa	192	−13.94	7.11		
		aa	1	−17.60	.		
	Total response	AA	3890	9.63	5.68	0.10	0.9026
		Aa	193	9.45	5.50		
		aa	1	8.60	.		
Right ear							
TEOAE amplitude (dB SPL)	1 kHz	AA	3668	−7.62	6.93	0.04	0.9637
		Aa	190	−7.59	6.86		
		aa	1	−5.80	.		
	2 kHz	AA	3727	−9.00	6.52	0.00	0.9970
		Aa	192	−9.00	6.18		
		aa	1	−9.50	.		
	3 kHz	AA	3722	−11.38	7.17	2.23	0.1074
		Aa	193	−12.42	6.90		
		aa	1	−6.00	.		
	4 kHz	AA	3716	−13.54	7.41	0.36	0.6982
		Aa	193	−13.85	7.28		
		aa	1	−8.80	.		
	Total response	AA	3738	10.50	5.66	0.17	0.8470
		Aa	192	10.29	5.39		
		aa	1	11.90	.		
Left ear							
Tympanometry	Middle ear compliance (cm^3^)	AA	4767	0.64	0.42	0.22	0.7987
		Aa	225	0.65	0.40		
		aa	2	0.55	0.07		
	Middle ear pressure (daPa)	AA	4776	−39.73	62.29	0.32	0.7292
		Aa	226	−37.96	57.50		
		aa	2	−10.00	14.14		
Right ear							
Tympanometry	Middle ear compliance (cm^3^)	AA	4768	0.62	0.42	1.60	0.2028
		Aa	228	0.67	0.44		
		aa	2	0.45	0.07		
	Middle ear pressure (daPa)	AA	4780	−39.37	61.61	0.14	0.8712
		Aa	227	−40.00	60.16		
		aa	2	−17.50	10.61		

**Table 3:** Effect of carrier (1) (*FLG* R501X or *FLG* 2282del4) versus non-carrier (0) status on 30 hearing variables as tested by one-way ANOVA tests.

		Status	N	Mean	SD	F	P
Right ear							
Air conduction hearing threshold (dB HL)	0.5 kHz	0	4716	6.18	7.21	0.62	0.4308
		1	455	6.46	7.32		
	1 kHz	0	4790	4.33	7.57	1.42	0.2333
		1	459	4.77	8.05		
	2 kHz	0	4789	4.02	7.26	0.14	0.7041
		1	459	4.15	7.41		
	4 kHz	0	4788	3.83	8.31	0.00	0.9926
		1	459	3.82	8.35		
	8 kHz	0	4700	9.03	10.09	0.02	0.8892
		1	450	9.10	9.63		
	Average 0.5, 1, 2, 4, 8 kHz	0	4694	5.44	6.28	0.56	0.4543
		1	449	5.67	6.56		
Left ear							
Air conduction hearing threshold (dB HL)	0.5 kHz	0	4718	6.48	7.31	0.04	0.8508
		1	455	6.42	6.90		
	1 kHz	0	4789	4.51	7.66	0.58	0.4454
		1	459	4.23	7.08		
	2 kHz	0	4787	4.21	7.50	0.02	0.8981
		1	459	4.26	7.25		
	4 kHz	0	4786	4.69	8.60	0.71	0.3990
		1	458	4.33	7.91		
	8 kHz	0	4699	9.18	10.35	0.86	0.3545
		1	449	8.71	10.13		
	Average 0.5, 1, 2, 4, 8 kHz	0	4697	5.77	6.27	0.36	0.5510
		1	448	5.59	6.20		
Bone conduction hearing threshold (dB HL)	0.5 kHz	0	4628	−1.25	6.47	0.05	0.8241
		1	446	−1.18	6.93		
	1 kHz	0	4765	−2.31	6.42	0.31	0.5772
		1	457	−2.13	7.19		
	2 kHz	0	4637	0.94	7.18	0.01	0.9038
		1	446	0.90	7.82		
	Average 0.5, 1, 2 kHz	0	4623	−0.90	5.28	0.14	0.7128
		1	446	−0.80	6.22		
Left ear							
TEOAE amplitude (dB SPL)	1 kHz	0	3677	−8.75	6.98	0.02	0.8939
		1	352	−8.70	7.08		
	2 kHz	0	3709	−10.14	6.73	0.45	0.5047
		1	355	−10.39	6.71		
	3 kHz	0	3704	−12.11	7.17	1.30	0.2538
		1	355	−12.56	6.83		
	4 kHz	0	3717	−13.80	7.59	0.58	0.4452
		1	355	−14.12	7.01		
	Total response	0	3727	9.64	5.70	0.31	0.5792
		1	356	9.46	5.45		
Right ear							
TEOAE amplitude (dB SPL)	1 kHz	0	3515	−7.66	6.91	1.46	0.2271
		1	343	−7.19	7.02		
	2 kHz	0	3574	−9.04	6.54	1.54	0.2153
		1	345	−8.58	6.16		
	3 kHz	0	3569	−11.39	7.19	1.60	0.2060
		1	346	−11.90	6.87		
	4 kHz	0	3565	−13.52	7.42	0.77	0.3797
		1	344	−13.89	7.19		
	Total response	0	3585	10.47	5.67	0.64	0.4242
		1	345	10.72	5.44		
Left ear							
Tympanometry	Middle ear compliance (cm^3^)	0	4562	0.64	0.42	1.64	0.2007
		1	431	0.66	0.44		
	Middle ear pressure (daPa)	0	4572	−39.71	62.01	0.06	0.8113
		1	431	−38.97	62.74		
Right ear							
Tympanometry	Middle ear compliance (cm^3^)	0	4565	0.62	0.42	2.19	0.1389
		1	432	0.65	0.44		
	Middle ear pressure (daPa)	0	4579	−39.44	61.49	0.02	0.8934
		1	429	−39.02	62.08		

There was no association with better versus worse hearing in either of the two mutations (data not shown). The analyses involving enhanced or diminished bone conduction did not show evidence of association with R501X (data not shown). A nominal association (P = 0.013) was found between the average bone conduction better versus worse hearing and 2282del4 but there was neither association nor evidence of any magnitude of difference by genotype for R501X. The analyses involving better versus worse cochlear function/middle ear transmission did not show evidence of association with R501X or 2282del4 (data not shown).

Table 4 shows the contingency tables for the phenotype bilateral middle ear effusion for both R501X and 2282del4. There was no relationship between *FLG* mutations and middle ear effusion. The analysis of association between cases with type A tympanograms (i.e. no middle ear effusion) and the hearing variables showed no evidence of association with either mutation.

**Table 4:** Effect of *FLG* R501X and 2282del4 on bilateral middle ear effusion.

		R501X		2282del4		
		AA	Aa	AA	Aa	aa
Bilateral A	Observed	3881	162	3856	186	2
	Expected	3874.00	169.00	3861.22	181.25	1.53
Bilateral B	Observed	62	2	63	1	0
	Expected	61.32	2.68	61.11	2.87	0.02
Bilateral C1	Observed	121	3	118	6	0
	Expected	118.82	5.18	118.40	5.56	0.05
Bilateral C2	Observed	58	5	61	2	0
	Expected	60.37	2.63	60.15	2.82	0.02
Unilateral B	Observed	164	8	168	4	0
	Expected	164.81	7.19	164.23	7.71	0.07
Other	Observed	780	41	783	38	0
	Expected	786.68	34.32	783.89	36.80	0.31
Pearson χ^2^		P = 0.402		P = 0.937		

Type A indicates normal middle ear function. Type B indicates middle ear effusion. Type C1 indicates slight negative middle ear pressure. Type C2 indicates negative middle ear pressure. Other indicates grommet, perforation, or type C2 tympanogram in at least one ear. Expected correspond to the expected contingency values were the null hypothesis is that the probabilities for each phenotype are independent of the mutation.

## Discussion

This work is the first analysis of two common filaggrin mutations (R501X and 2282del4) for possible effect on hearing phenotypes. It was undertaken in a large (N = 5,377) cohort of UK children. These children were analysed in detail for a large number of hearing variables when they were nine years old. We did not find any support for the hypothesis that *FLG* mutation carrier status, via known expression in the tympanic membrane, might affect hearing. Two instances of nominal association were found in our study. However, both instances may be the result of type-I error, since we conducted 140 tests. The correction for multiple testing results in a limit of P = 0.0004 to reject the null hypothesis for an α = 0.05, a limit well below the minimum P value observed in our study. Furthermore, these nominal associations were not consistent between the two different *FLG* mutations.

Our study was well powered (N = 5,377). This reduces the chance of false negatives. Therefore, our conclusions of no effect of R501X and 2282del4 on hearing thresholds or on otoacoustic emissions are robust. The same applies to our results indicating no effect of *FLG* mutations on tympanic membrane compliance, middle ear pressure or incidence of middle ear effusion. However, our results derive from a UK sample of nine year old children. It remains possible that there might be genotype-dependent effects in other age ranges and populations.
